# Supplementary material for: The role of toxin/antidote genes in the maintenance and evolution of accessory chromosomes in Fusarium
Source: Genetics. 2025 Sep 19;231(4):iyaf197. doi: 10.1093/genetics/iyaf197 (PMC12693581; doi:10.1093/genetics/iyaf197)
Supplement: iyaf197_Supplementary_Data [file iyaf197_supplementary_data.zip › Supplementary_Figures_and_Captions_GENETICS-2025-308521.pdf]

The role of toxin/antidote genes in the maintenance and evolution of accessory chromosomes in *Fusarium*.

Linnea Sandell, Adrian Forsythe, Anna Mirandola, Samuel Jorayev, Andrew S. Urquhart, Alexandra Granger Farbos, Sven J. Saupe & Aaron A. Vogan

Supplementary Figures

1 Distribution of Spok genes across species and *F. oxysporum forma specialis* 2

2 Phylogeny of FuSpok genes from *Fusarium oxysporum* 3

3 *Spok3* killing assay using *S. cerevisiae* 4

4 *FuSpok* killing assay. 5

Supplementary Table captions **6**

Supplementary File captions **6**

Supplementary Figures

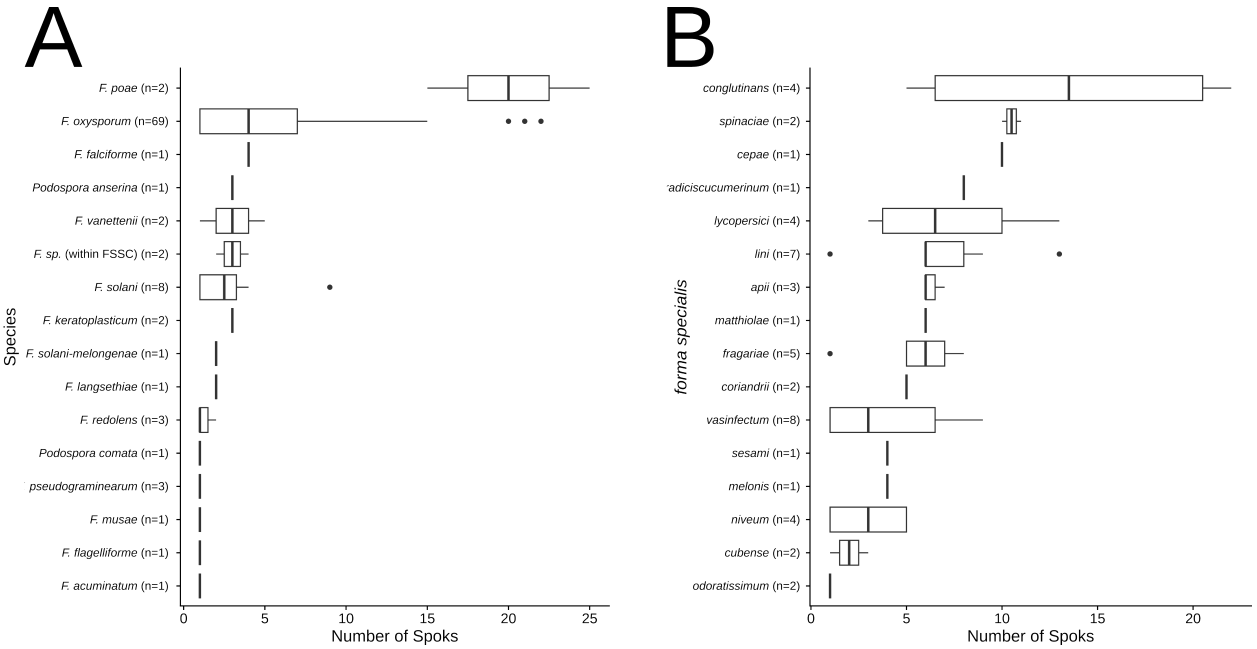

Supplementary Figure 1: The number of Spok genes found across (A) *Fusarium* species and different *F. oxysporum forma specialis*. The number of genomes that were considered in the search for *Spok* genes is included beside the species/*forma specialis* name.

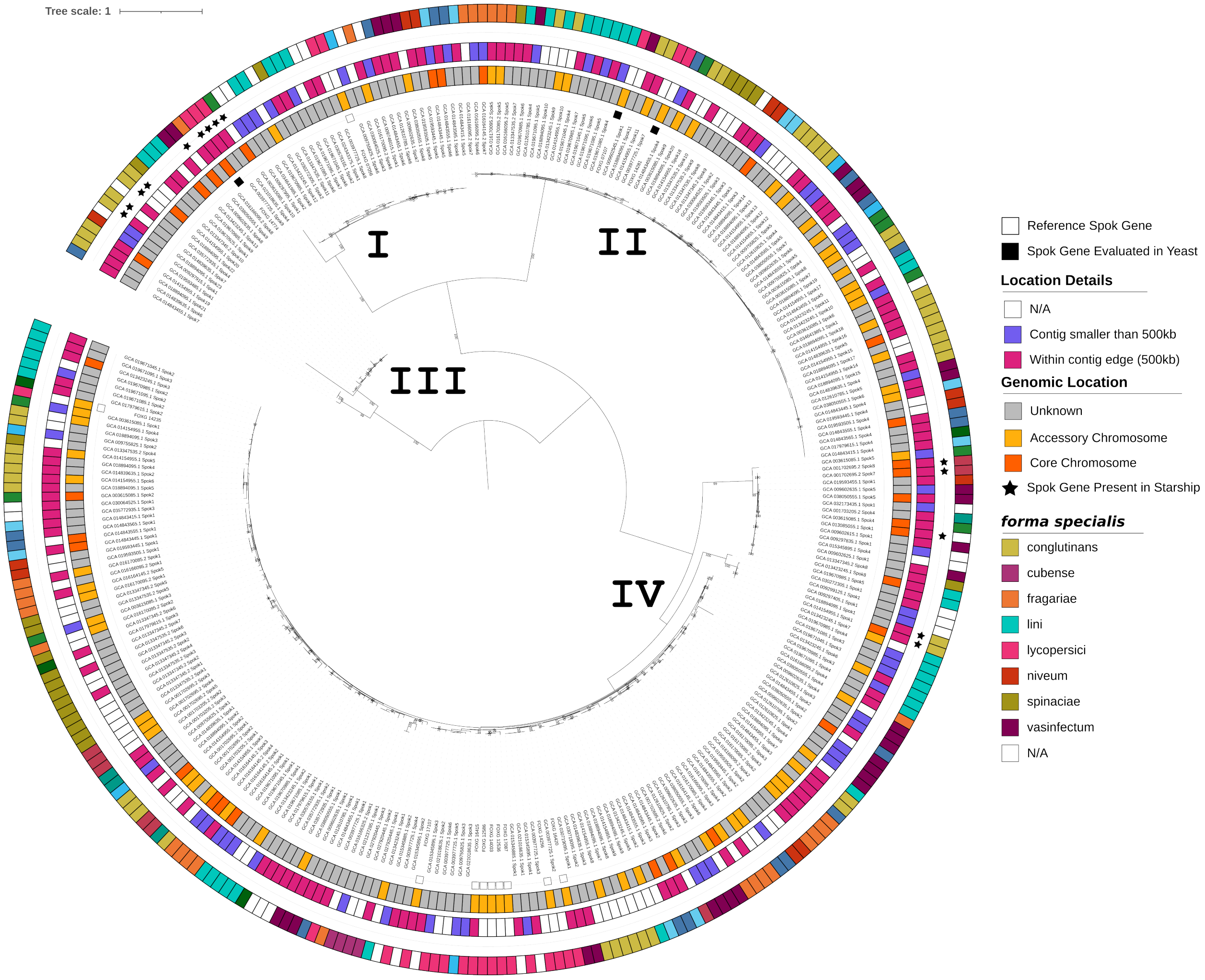

Supplementary Figure 2: Maximum-liklihood phylogeny (mid-point rooted) of *Spok* gene homologs from *Fusarium oxysporum*. The support values are ultrafast bootstrap (n=1000) approximations from `IQ-tree`, with only bootstraps with a value of $>= 95$ shown. Tips annotated with a black square indicate *FuSpok* genes that are part of the reference set (**Supplementary Table 2**), with filled squares denoting the *FuSpok* genes which were assayed in this study (**Supplementary Table 4**). The inner coloured ring contains annotations of the genomic locations of *FuSpok* genes, while the outer coloured ring depicts the *forma specialis* designations for *F. oxysporum* genomes. In addition, we included annotations with stars to denote *FuSpoks* which were found inside of *Starship* elements.

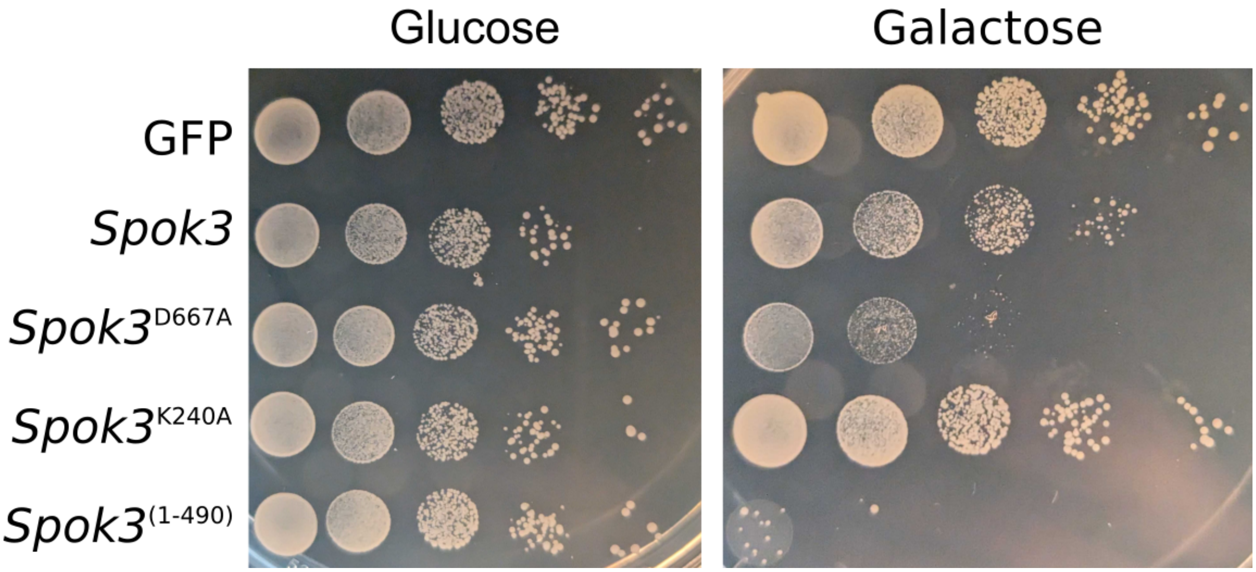

Supplementary Figure 3: Killing assay with wild type and mutated versons of the *P. anserina Spok3* gene expressed in *S. cerevisiae*

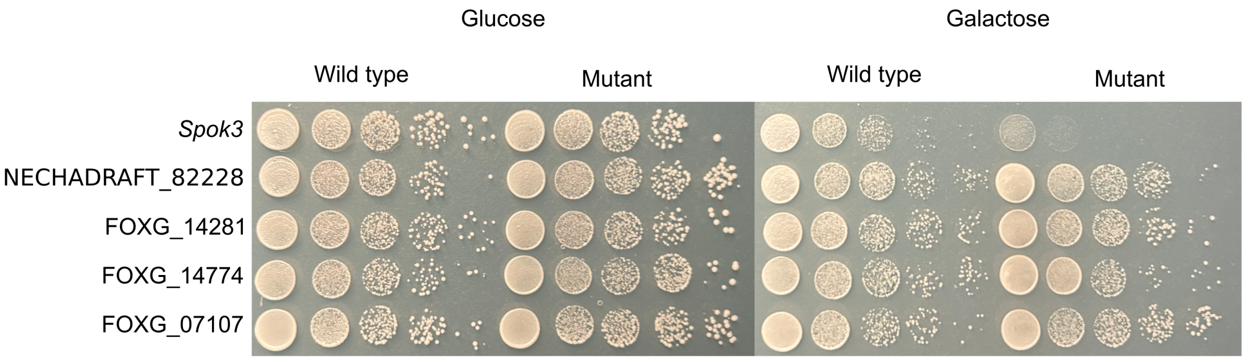

Supplementary Figure 4: Killing assay of various *FuSpok* genes that showed no phenotypic effects when expressed in *S. cerevisiae*. Mutant refers to strains with vectors containing *FuSpok* homologs with a D to A mutation in the active site of the resistance domain. See **Supplementary Table 4** for details.

Supplementary Table captions

Supplementary Table 1: We identified *Spok* homologs from 146 *Fusarium* genome assemblies collected from NCBI. Each genome assembly was assigned a unique genome code (the ”ome” codes from the mycotools database), generated using the first 3 letters of genus and species names, plus a numerical identifier.

Supplementary Table 2: The final set of *Spok* homologs and their genomic coordinates, identified within the genomes listed in Table 1 and additional reference genes.

Supplementary Table 3: Primer sequences for amplification of *FuSpok* genes.

Supplementary Table 4: Details of *Spok* homolosg investigated in this study, including specific site mutations from site-directed mutagenesis experiment conducted on *FuSpoks* in yeast.

Supplementary File captions

Supplementary File 1: Fasta file of entire plasmid P001 sequence.

Supplementary File 2: Multiple sequence alignment of all *Spok* homologs. Used for generating phylogenetic trees.
